# Shell characterization of the youngest valid species of the European Eocene genus *Neochelys* (Pleurodira, Podocnemididae): The Spanish Bartonian *Neochelys salmanticensis*

**DOI:** 10.1002/ar.25225

**Published:** 2023-04-18

**Authors:** Adán Pérez‐García, Andrea Guerrero, Santiago Martín de Jesús, Francisco Ortega

**Affiliations:** ^1^ Grupo de Biología Evolutiva, Facultad de Ciencias, Departmento de Física Matemática y de Fluidos UNED Madrid Spain; ^2^ Colección de Vertebrados Fósiles de la Cuenca del Duero (Sala de las Tortugas), Facultad de Ciencias, Departamento de Geología Universidad de Salamanca Salamanca Spain

**Keywords:** anatomy, Duero Basin, Erymnochelyinae, middle Eocene, Pelomedusoides, Salamanca Province, variability

## Abstract

The freshwater pleurodiran turtle *Neochelys* is the best‐represented member of Podocnemididae in the European record, being known by eight Eocene species. The youngest of them is the Bartonian (middle Eocene) *Neochelys salmanticensis*, from the Duero Basin (Salamanca Province, Central Spain). It corresponds to the largest representative known for this genus, its shell reaching 50 cm in length. Despite this form was defined several decades ago, the information currently available on it is very limited, being restricted to shell remains of less than 10 individuals. In fact, this species lacks a valid diagnosis, considering the current knowledge about the genus. Numerous remains (i.e., more than 1,200) of the shell of this Spanish species are identified. Its detailed study is presented here, so that the anatomy of its shell is characterized in detail. In addition, several aspects related to its intraspecific variability are analyzed, relative to the individual, ontogenetic, and sexual variability. In this way, the shell of *N. salmanticensis* can be characterized with greater precision than that of any other species of the genus.

## 1 INTRODUCTION

**FIGURE 1:**
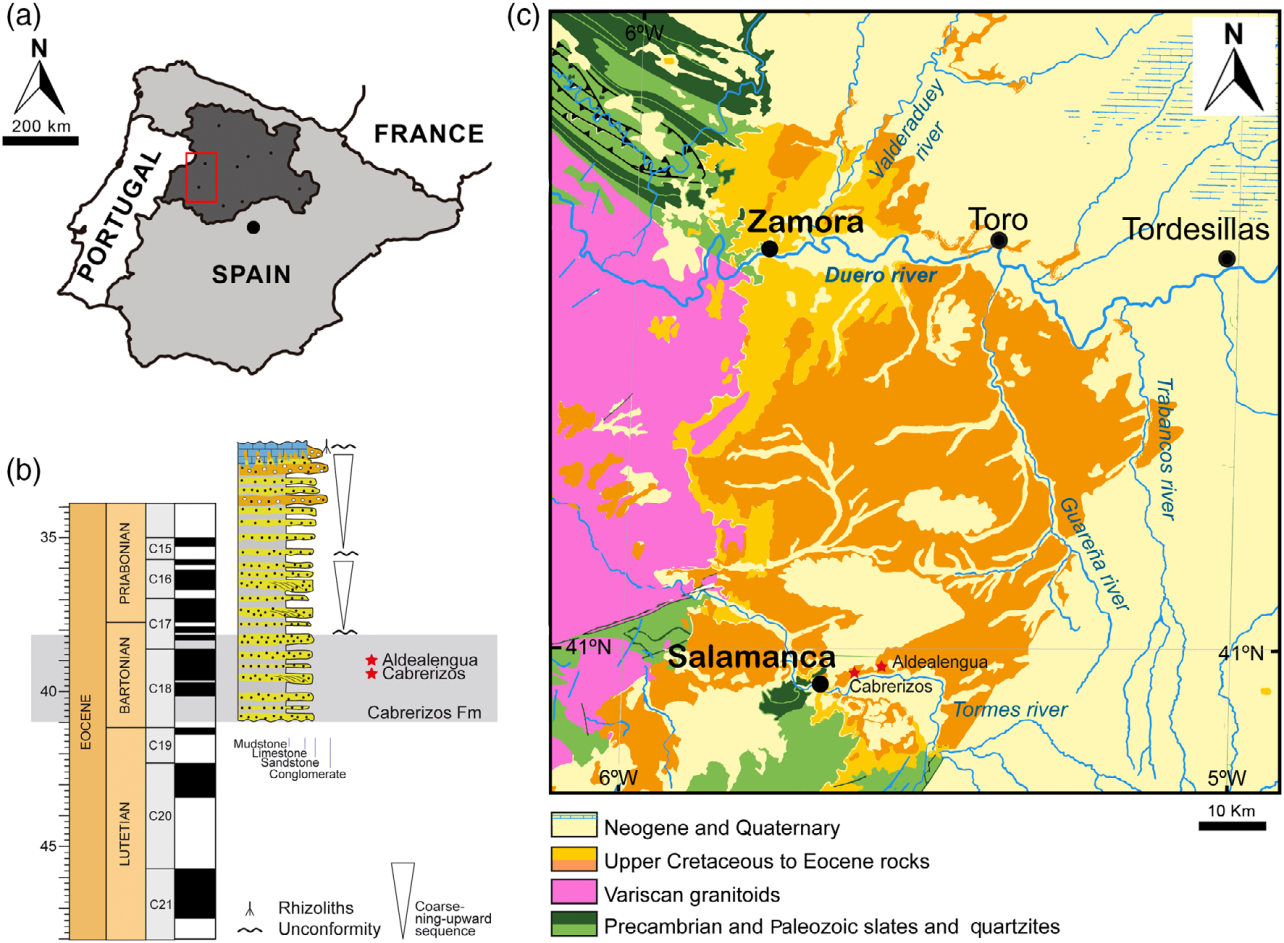
Geological context of the Spanish Bartonian (middle Eocene) podocnemidid turtle *Neochelys salmanticensis* occurrences. (a) Southwestern sector of the Duero Basin (red square), located at the southwestern region of the Autonomous Community of Castile and Leon (dark gray). (b) Chronostratigraphic location of the Cabrerizos (Teso de la Flecha and Caenes) and Aldealengua fossil sites (red stars). (c) Geological map of the southwestern sector of the Duero Basin (red square in a) showing the location of the Cabrerizos and Aldealengua fossil sites (red stars; modified from Ortega et al., 2022)

The pan‐pleurodiran turtles are relatively abundant and diverse in the Upper Cretaceous record of Europe, being represented by both members of Dortokidae (stem Pleurodira) and Bothremydidae (crown group Pleurodira) see Pérez‐García, 2017, and references therein). Pan‐Pleurodira is not recognized in the Paleocene record of this continent (Pérez‐García, 2020). In fact, this lineage is poorly represented in the Cenozoic record of Europe, except for the Eocene record, in which two Podocnemidoidea lineages are recognized: Bothremydidae and Podocnemididae (Pérez‐García, 2018). The best‐represented lineage is that of the podocnemidids, being known through two European genera distributed between the lower and the upper Eocene. One of them is a coastal form, with a less abundant and diverse record: *Eocenochelus* (Pérez‐García et al., 2017). The other is *Neochelys*, a freshwater taxon identified in several countries, through several species (Pérez‐García & de Lapparent de Broin, 2013). The largest concentration of *Neochelys* remains currently identified comes from the Duero Basin (Castile and Leon, Spain), including about 4,500 inventoried remains that are part of the Collection of Fossil Vertebrates of the Duero Basin (Sala de las Tortugas) of the Universidad de Salamanca (see Ortega et al., 2022, and references therein). They correspond to complete shells and shell remains (i.e., partial shells and articulated and isolated plates), but also to skulls, vertebrae, girdle elements, and appendicular bones, mostly unpublished. Some of them have been found as disarticulated and isolated remains, but others are recognized as articulated elements, even corresponding to relatively complete skeletons. The *Neochelys* remains from the Duero Basin come from middle and upper Eocene levels, from the Lutetian to the Priabonian. Two valid species are recognized there, but a greater diversity is represented (Ortega et al., 2022; Pérez‐García, 2017). The first described species was *Neochelys salmanticensis*, from the Bartonian (middle Eocene) of Cabrerizos (Salamanca Province, Central Spain; Figure 1). It is the largest currently known species of the genus, as well as the youngest. As with most *Neochelys* species, only shell elements of *N. salmanticensis* have been published. However, despite the abundance of remains found in its type area, very few have been figured or described so far, most of them having been analyzed decades ago (in the late 1960s and early 1970s; see Jiménez Fuentes, 1968, 1970a, 1970b, 1970c, 1971). An updated diagnoses for this species, as well as its complete shell anatomical characterization considering the current knowledge about the genus *Neochelys*, have not yet been carried out.

**FIGURE 2:**
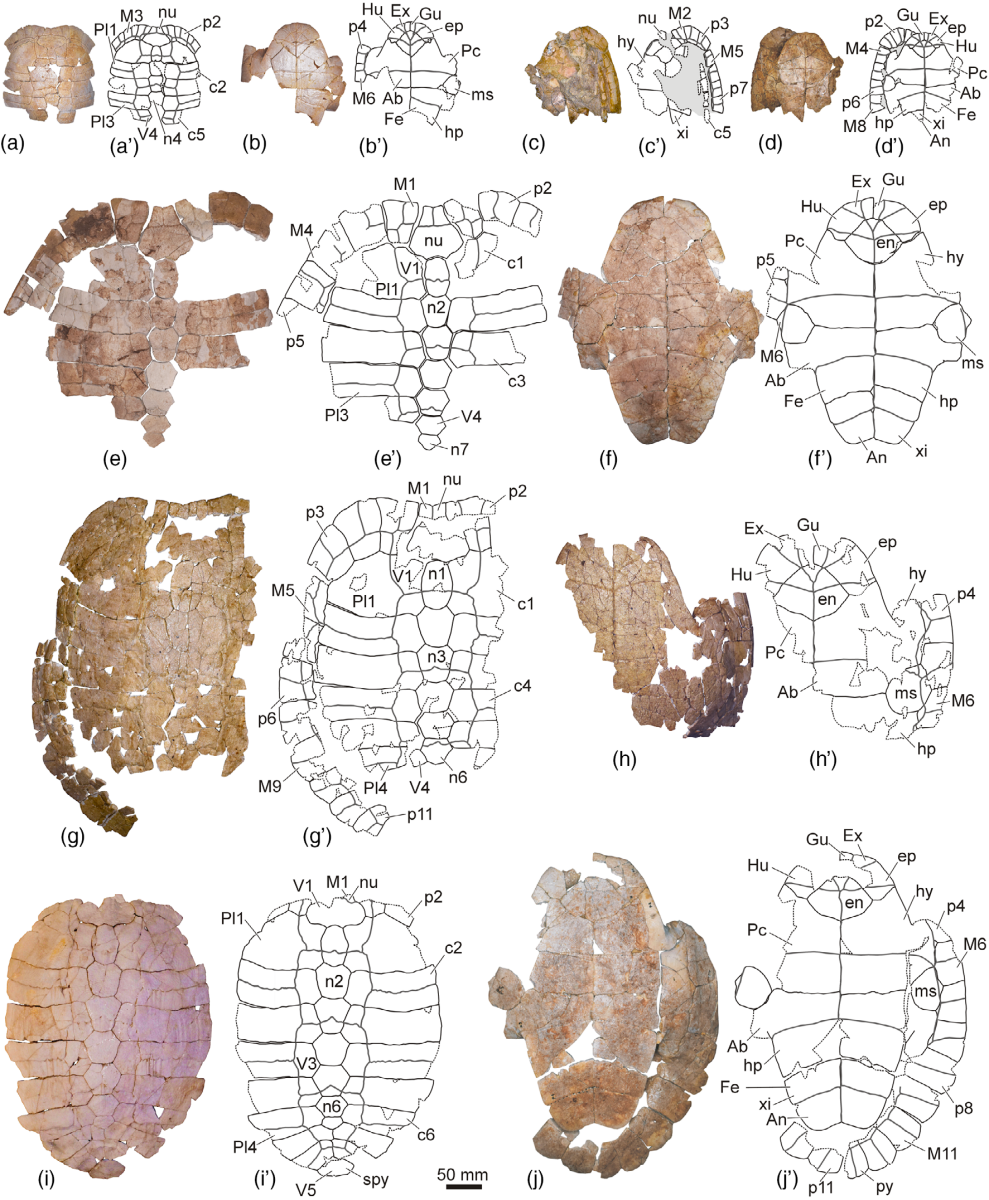
Shells of the Spanish podocnemidid turtle *Neochelys salmanticensis*, from the Bartonian (middle Eocene) of Cabrerizos (Salamanca Province), in dorsal (left) and ventral (right) views. (a, b) STUS 12152 from Teso de la Flecha; (c, d) STUS 9302 from Caenes; (e, f) STUS 617 from Caenes; (g, h) STUS 14283 from Teso de la Flecha; (i, j) STUS 263 from Teso de la Flecha

**FIGURE 3:**
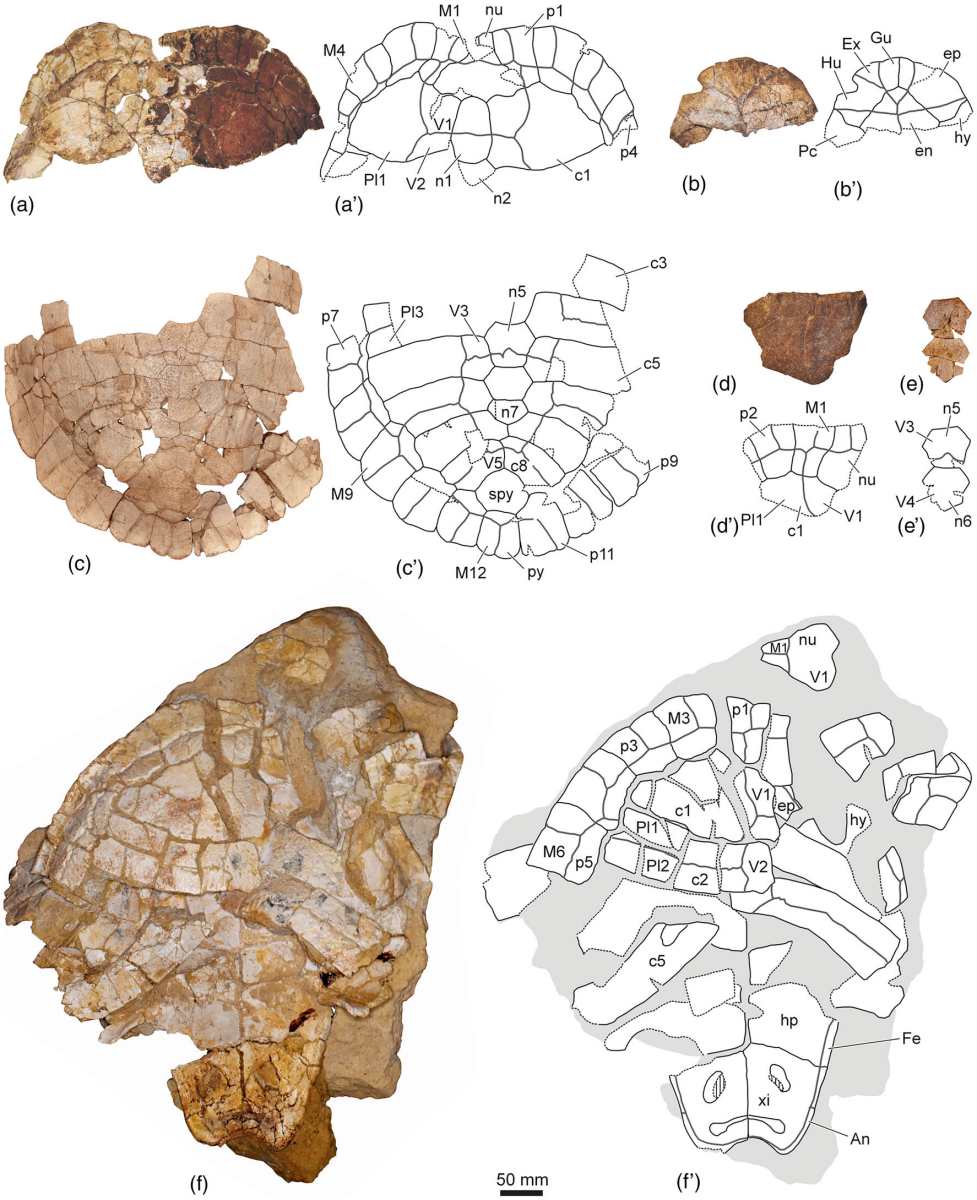
Shells and articulated carapace remains of the Spanish podocnemidid turtle *Neochelys salmanticensis*, from the Bartonian (middle Eocene) of Cabrerizos (Salamanca Province), in dorsal or dorsal (left) and ventral (right) views. (a, b) STUS 209 from Caenes; (c) STUS 12147 from Teso de la Flecha; (d) STUS 1605 from Teso de la Flecha; (e, f) STUS 12153 from Caenes

**FIGURE 4:**
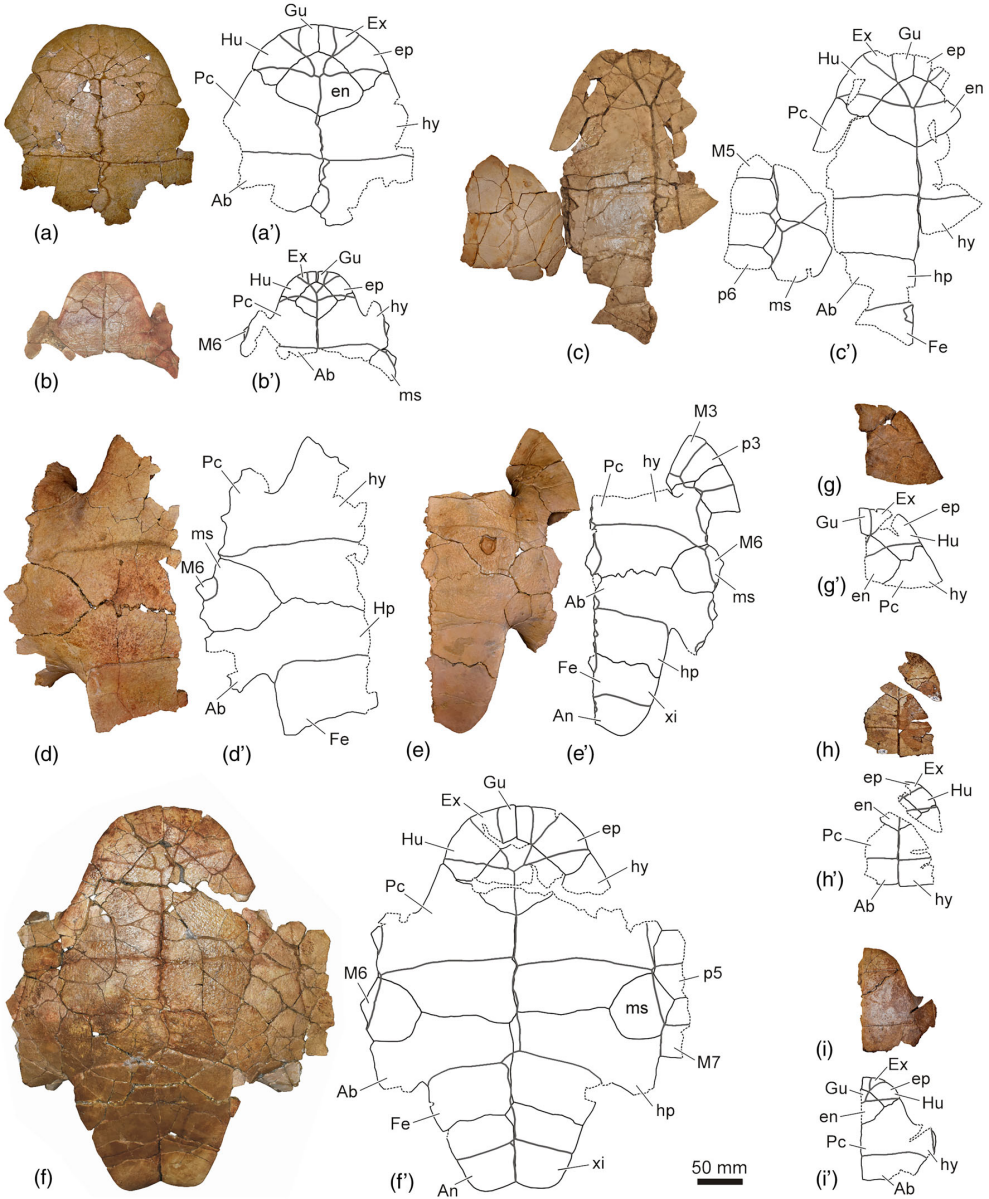
Plastra of the Spanish podocnemidid turtle *Neochelys salmanticensis*, from the Bartonian (middle Eocene) of Cabrerizos (Salamanca Province), in ventral view. (a) STUS 180 from Caenes; (b) STUS 12150 from Caenes; (c) STUS 245, from Caenes; (d) STUS 14392 from Caenes; (e) STUS 12148 from Teso de la Flecha; (f) STUS 14359 from Teso de la Flecha; (g) STUS 182 from Caenes; (h) STUS 625 from Caenes; (i) STUS 311 from Caenes

**FIGURE 5:**
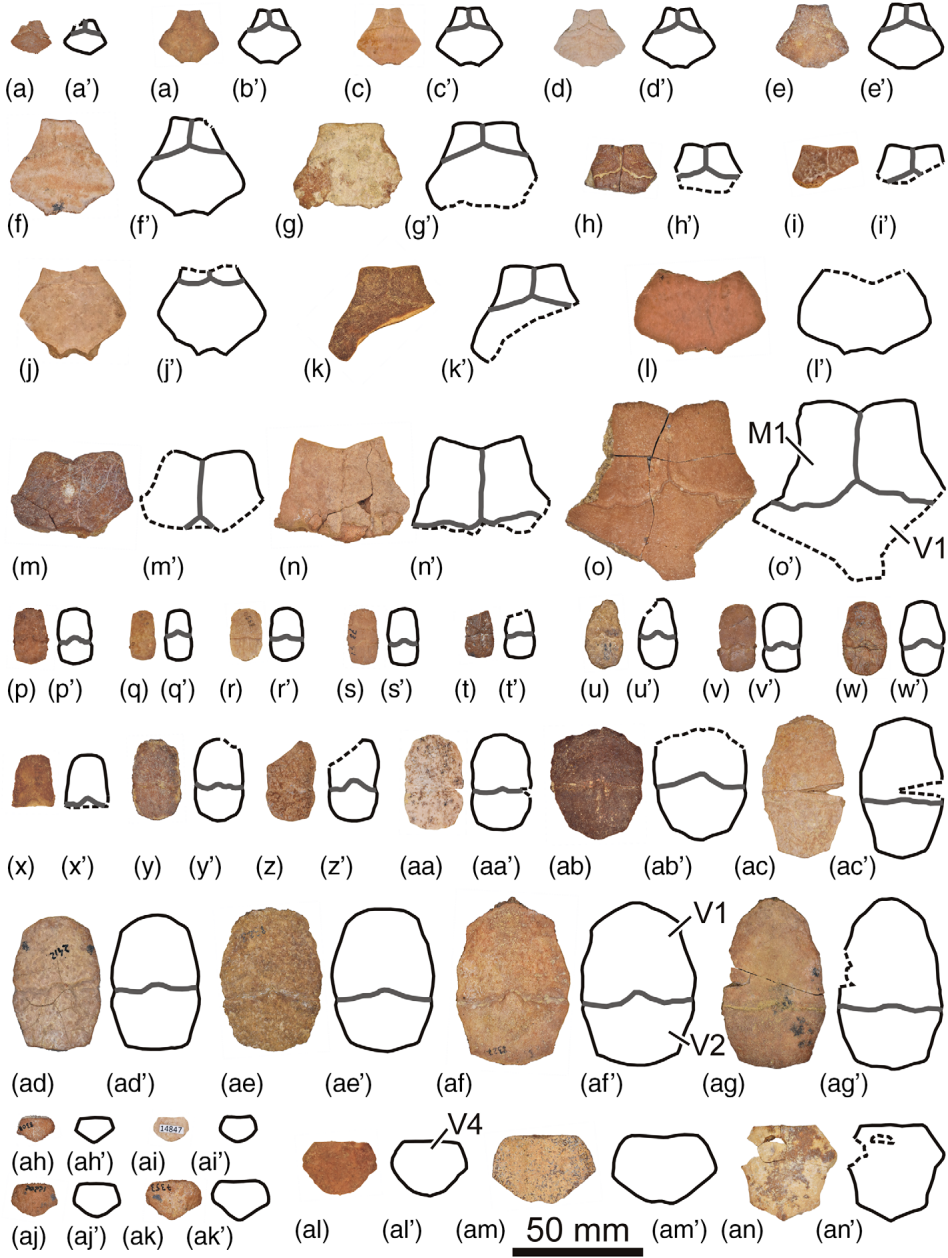
Nuchals (a–o), first neurals (p–ag) and last neurals (ah–an) of the Spanish podocnemidid turtle *Neochelys salmanticensis* from the Bartonian (middle Eocene) of Cabrerizos (Salamanca Province), in dorsal view. (a) STUS 13544; (b) STUS 8300; (c) STUS 8267; (d) STUS 13162; (e) STUS 12505; (f) STUS 13410; (g), STUS 12506; (h) STUS 8166; (i) STUS 8232; (j) STUS 8194; (k) STUS 8165; (l) STUS 7470; (m) STUS 14177; (n) STUS 8200; (o) STUS 12980; (p) STUS 13178; (q) STUS 8331; (r) STUS 8330; (s) STUS 7351; (t) STUS 7350; (u) STUS 8332; (v) STUS 14205; (w) STUS 8329; (x) STUS 8319; (y) STUS 8314; (z) STUS 8112; (aa) STUS 13446; (ab) STUS 7358; (ac) STUS 8142; (ad) STUS 2412; (ae) STUS 8328; (af) STUS 8327; (ag) STUS 12706; (ah) STUS 8308; (ai) STUS 14847; (aj) STUS 12202; (ak) STUS 7353; (al) STUS 8260; (am) STUS 8160; (an) STUS 8159. All of them come from Caenes except STUS 14177 (m) and STUS 8314 (y) from Teso de la Flecha

**FIGURE 6:**
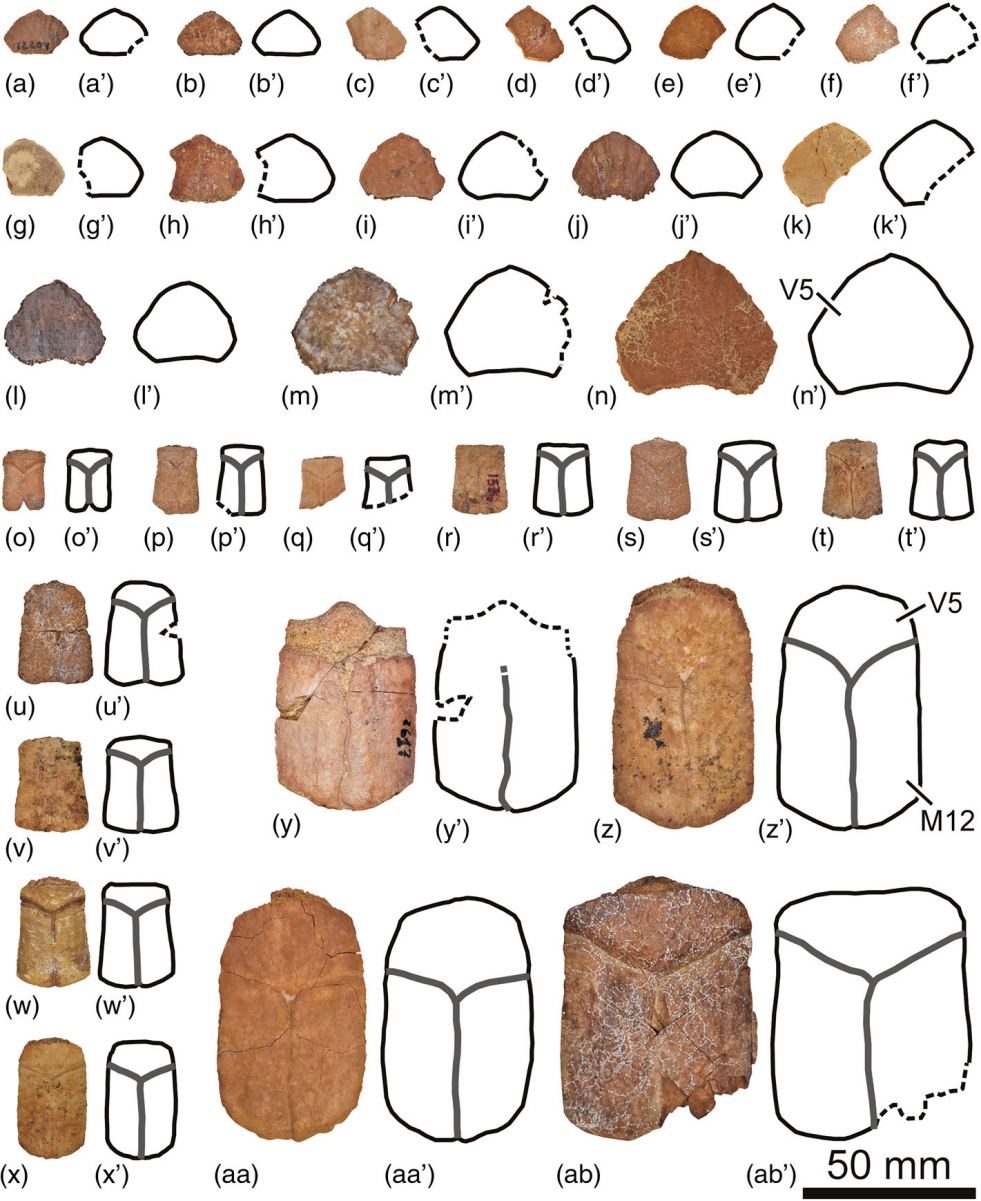
Suprapygals (a–n) and pygals (o–ab) of the Spanish podocnemidid turtle *Neochelys salmanticensis* from the Bartonian (middle Eocene) of Cabrerizos (Salamanca Province), in dorsal view. (a) STUS 12204; (b) STUS 2512; (c) STUS 7489; (d) STUS 8226; (e) STUS 8167; (f) STUS 13498; (g) STUS 8303; (h) STUS 7490; (i) STUS 13161; (j) STUS 16642; (k) STUS 8156; (l) STUS 18302; (m) STUS 8183; (n) STUS 8250; (o) STUS 8321; (p) STUS 8322; (q) STUS 7474; (r) STUS 1574; (s) STUS 8175; (t) STUS 8323; (u) STUS 8324; (v) STUS 8119; (w) STUS 8325; (x) STUS 7473; (y) STUS 2617; (z) STUS 8242; (aa) STUS 8241; (ab) STUS 8326. All of them come from Caenes

**FIGURE 7:**
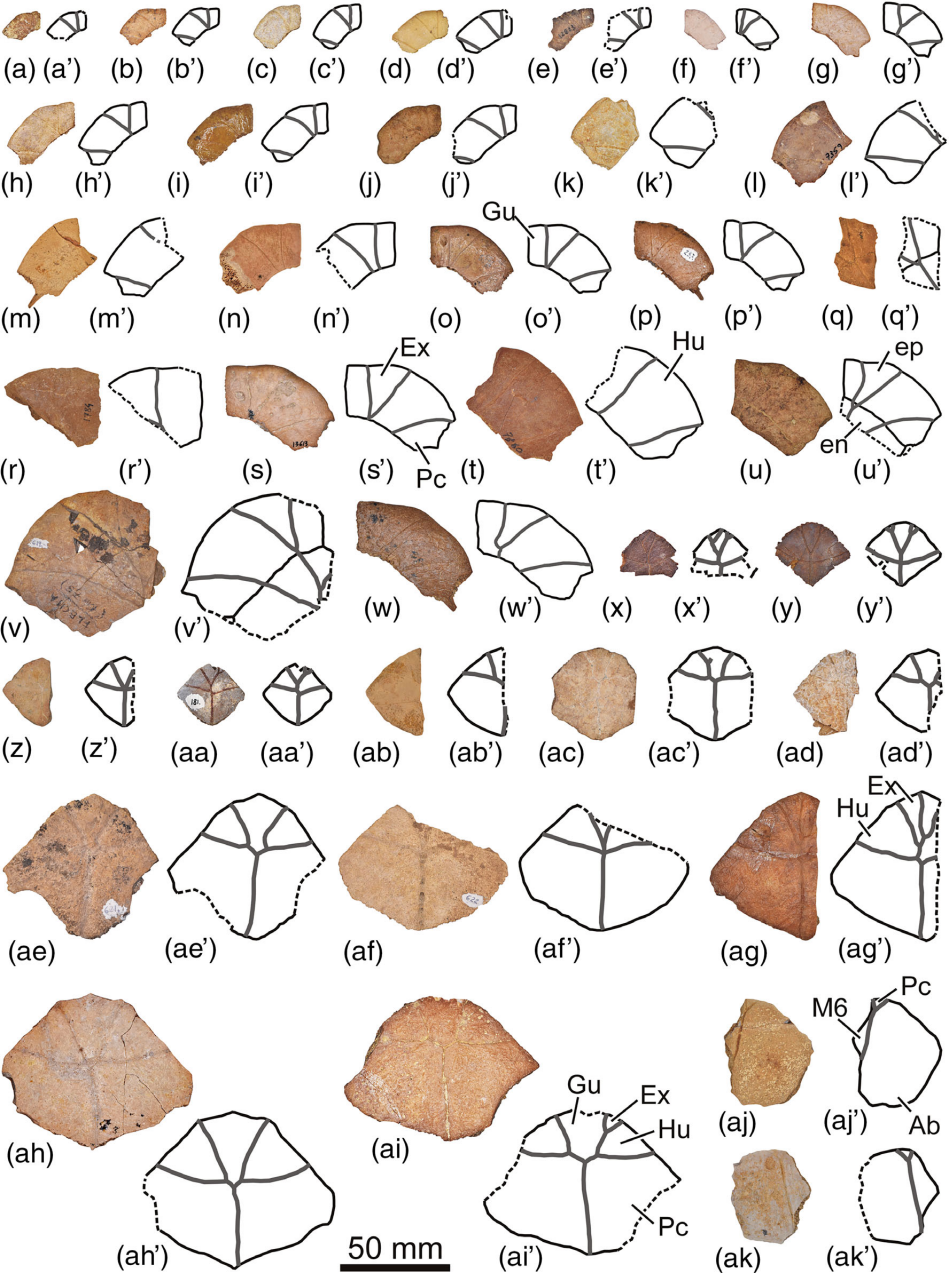
Epiplastra (a–t, w), articulated epiplastra and entoplastra (u–v), entoplastra (x–ai), and xiphiplastra (aj–ak) of the Spanish podocnemidid turtle *Neochelys salmanticensis* from the Bartonian (middle Eocene) of Cabrerizos (Salamanca Province), in ventral view. (a) STUS 8304; (b) STUS 8291; (c) STUS 8299; (d) STUS 13159; (e) STUS 12833; (f) STUS 8131; (g) STUS 8157; (h) STUS 1905; (i) STUS 8333; (j) STUS 8794; (k) STUS 1914; (l) STUS 7359; (m) STUS 8116; (n) STUS 8164; (o) STUS 8334; (p) STUS 237; (q) STUS 8317; (r) STUS 1784; (s) STUS 13613; (t) STUS 7360; (u) STUS 12502; (v) STUS 619; (w) STUS 13158; (x) STUS 652; (y) STUS 13513; (z) STUS 13002; (aa) STUS 181; (ab) STUS 8129; (ac) STUS 13575; (ad) STUS 8182; (ae) STUS 621; (af) STUS 622; (ag) STUS 7362; (ah) STUS 13540; (ai) STUS 13153; (aj) STUS 8335; (ak) STUS 13308. All of them come from Caenes except STUS 8182 (ad) from Teso de la Flecha

**FIGURE 8:**
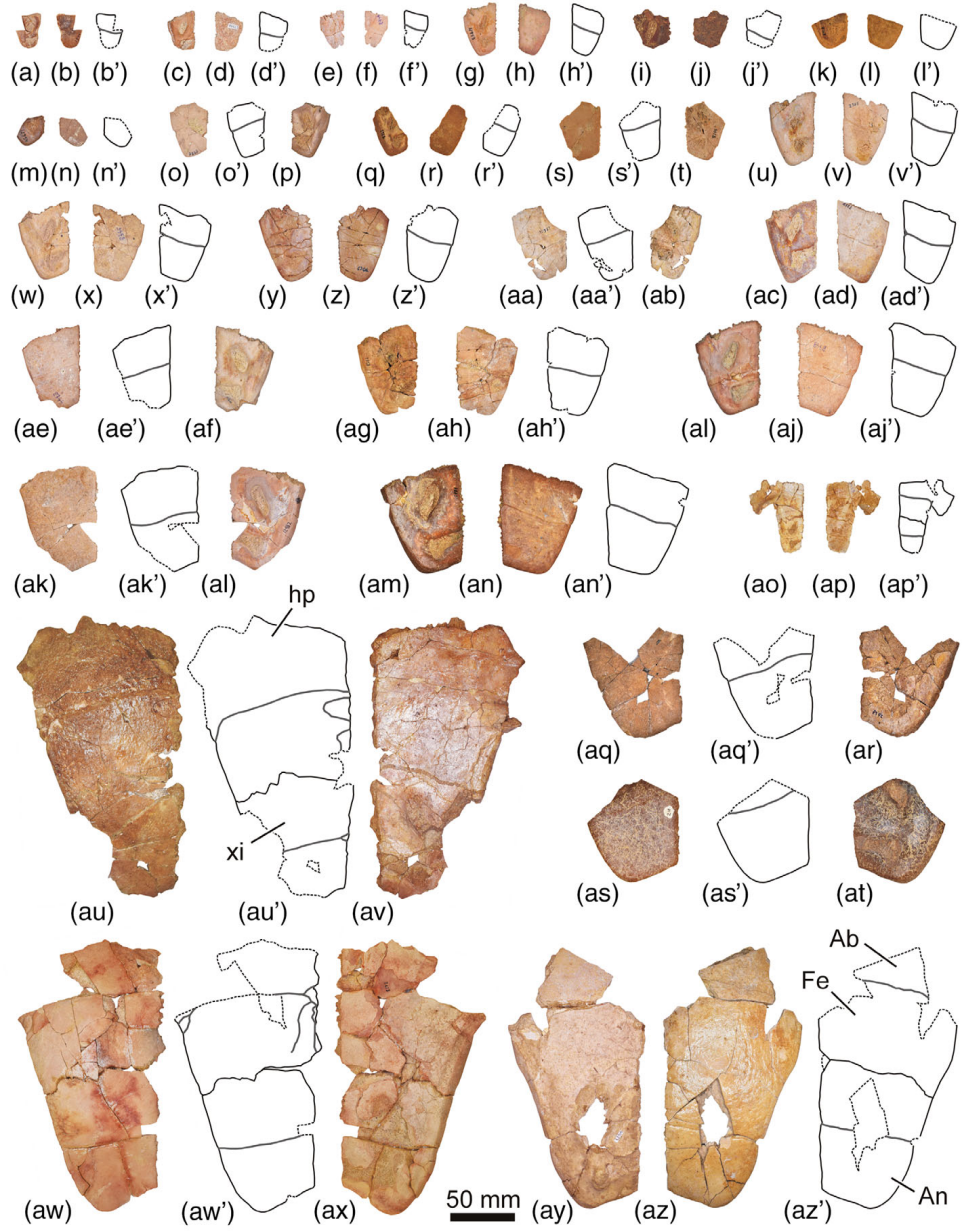
Xiphiplastra (a–an, aq–at) and articulated hypoplastra and xiphiplastra (ao, ap, au–az) of the Spanish podocnemidid turtle *Neochelys salmanticensis* from the Bartonian (middle Eocene) of Cabrerizos (Salamanca Province), in dorsal and ventral views. (a, b) STUS 7344; (c, d) STUS 17348; (e, f) STUS 8346; (g, h) STUS 12953; (i, j) STUS 8147; (k, l) STUS 8268; (m, n) STUS 12954; (o, p) STUS 7337; (q, r) STUS 8262; (s, t) STUS 8255; (u, v) STUS 2365; (w, x) STUS 8345; (y, z) STUS 2366; (aa, ab) STUS 13626; (ac, ad) STUS 7335; (ae, af) STUS 8344; (ag, ah) STUS 8253; (ai, aj) STUS 8343; (ak, al) STUS 12982; (am, an) STUS 12707; (ao, ap) STUS 8342; (aq, ar) STUS 8356; (as, at) STUS 210; (au, av) STUS 8320; (aw, ax) STUS 8272; (ay, az) STUS 12149. All of them come from Caenes except STUS 17348 (c, d) from Teso de la Flecha

The abundant collection of complete and partial shells, and articulated and isolated plates of *Neochelys* from the Bartonian of Cabrerizos (Figure 1), deposited in the Collection of Fossil Vertebrates of the Duero Basin (Sala de las Tortugas) of the Universidad de Salamanca, including the scarce specimens published up to now and numerous unpublished remains, has been analyzed in detail by us. *N. salmanticensis* is recognized as the best represented member of this genus, but also as the pleurodiran species with the largest number of known remains for the entire European Cenozoic record. A selection of specimens that allow characterizing in detail the shell of this species (the shell being the only comparable element among all the species of the genus, considering the availability of material) is presented here (Figures 2, 3, 4, 5, 6, 7, 8), so that an amended diagnosis for the taxon is proposed. In addition, the availability of numerous specimens allows us to provide data on its intraspecific variability, in relation to the ontogeny, individual variability, and sexual dimorphism.

*Institutional abbreviations*: MNHN.RA, Collection of Reptiles and Amphibians, Muséum national d'Histoire naturelle, Paris, France; MNHN.ZA.AC, Collection of Reptiles, Comparative Anatomy, Muséum national d'Histoire naturelle, Paris, France; STUS, Collection of Fossil Vertebrates of the Duero Basin (Sala de las Tortugas) of the Universidad de Salamanca, Salamanca, Spain.

*Anatomical abbreviations*: Ab, abdominal; An, anal; c, costal; Ex, extragular; en, entoplastron; ep, epiplastron; Fe, femoral; Gu, gular; hp, hypoplastron; Hu, humeral; hy, hyoplastron; M, marginal; ms, mesoplastron; n, neural; nu, nuchal; p, peripheral; Pc, pectoral; Pl, pleural; py, pygal; spy, suprapygal; V, vertebral; xi, xiphiplastron.

## 2 BACKGROUND ON THE KNOWLEDGE ABOUT *N. SALMANTICENSIS*

Jiménez Fuentes (1968) defined the species “*Stereogenys*” *salmanticensis* from the anterior region of a plastron (STUS 180; see figs. 4–6 in Jiménez Fuentes, 1968; Figure 4a) from the Bartonian of Teso de la Flecha (Cabrerizos, Salamanca Province, Spain). It was the first pleurodiran taxon defined for the Spanish record (Pérez‐García, 2017). In addition, Jiménez Fuentes (1968) identified an isolated entoplastron, also from the Duero Basin, but from the Lutetian of Corrales (Zamora Province, Spain; STUS 124; see figs. 7 and 8 in Jiménez Fuentes, 1968) as attributable to the same species. Jiménez Fuentes (1968) diagnosed the new species considering its relatively large size, as well as by the morphology and arrangement of some scutes (anteriorly widened gular, humerals lacking medial contact between them, humerals overlapping the entoplastron, extragulars close to or in contact with the entoplastron, sinuous sagittal sulcus) and plates (entoplastron wider than long) of the anterior plastral lobe.

**FIGURE 9:**
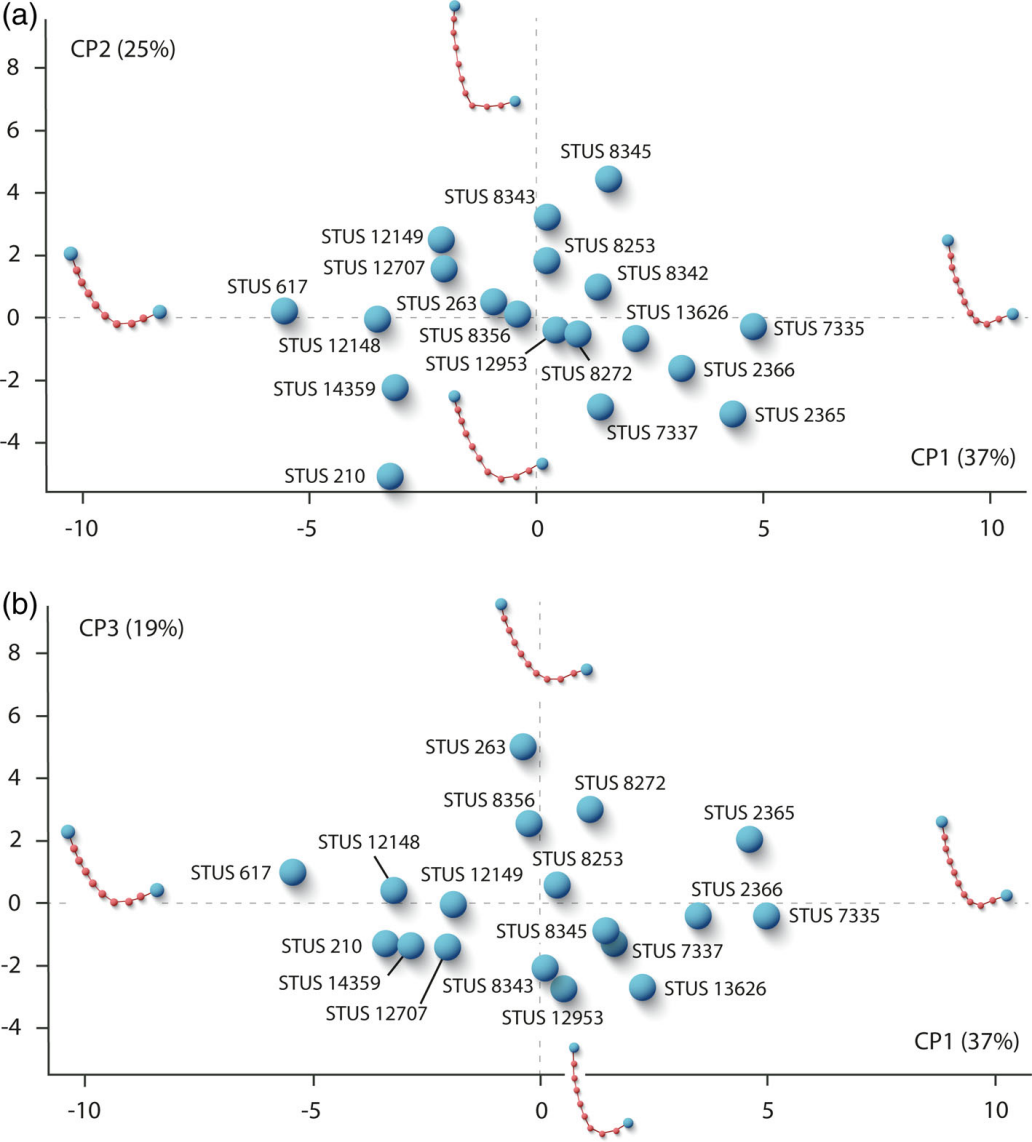
Shape differences of the xiphiplastron of the Spanish podocnemidid turtle *Neochelys salmanticensis* from the Bartonian (middle Eocene) of Cabrerizos (Salamanca Province). (a) Principal component 1 (*x*) and 2 (*y*). (b) Principal component 1 (*x*) and 3 (*y*)

Two years after the publication of the species “*Stereogenys*” *salmanticensis*, in 1970, Jiménez Fuentes attributed new remains to it: a partial plastron from the type locality and horizon (Bartonian of Teso de la Flecha), STUS 245 (see fig. 1 and plate 1 in Jiménez Fuentes, 1970a; Figure 4c). Its analysis allowed him to incorporate new characters into the diagnosis of the species, including mesoplastra smaller than the entoplastron and pectoral–abdominal sulci laterally overlapping the mesoplastra (Jiménez Fuentes, 1970a). The next specimen attributed to “*Stereogenys*” *salmanticensis* was the first relatively complete carapace from the type locality and horizon (STUS 263; see figs. 1–7 in Jiménez Fuentes, 1970b; Figure 2i,j). Thanks to this find, Jiménez Fuentes (1970b) included some new characters to the specific diagnosis (e.g., anal notch defining an obtuse angle; and presence of seven neurals, reaching the seventh pair of costals) and recognized one of the previously proposed characters as variable (presence or absence of overlapping of the pectoral scutes on the mesoplastra). Jiménez Fuentes (1970c) assigned three new specimens from the type locality and horizon to the species. They corresponded to two partial plastral lobes (STUS 311, see figs 1A, 2, 3 in Jiménez Fuentes, 1970c, and Figure 4i; STUS 182, see figs 1B and 4 in Jiménez Fuentes, 1970c, and Figure 4g) and one epiplastron (STUS 237, see figs. 1C and 5 in Jiménez Fuentes, 1970c, and Figure 7p). In addition, he assigned a mesoplastron from the Lutetian of Corrales (Zamora Province) to “*Stereogenys*” cf. *salmanticensis* (STUS 55, see figs. 1D and 6 in Jiménez Fuentes, 1970c). Considering the characters available in those specimens, Jiménez Fuentes (1970c) provided new data on the intraspecific variability of the taxon (i.e., the entoplastron can be slightly wider than long, and the ratio between the width of the anterior and posterior margins of the gular scute could be less than that of the holotype). A putative new taxon of Pleurodira from the Bartonian of Teso de la Flecha was described in that paper, from a single entoplastron (STUS 181; see figs. 7–9 in Jiménez Fuentes, 1970c; Figure 7aa): “*Podocnemis entodermica*.” It was defined as a small turtle that displayed a medial contact between the humerals, avoiding the contact between the gular and the pectorals, and showing the overlapping of the extragulars on the entoplastron (Jiménez Fuentes, 1970c).

“*Stereogenys*” *salmanticensis* and “*Podocnemis entodermica*” were not the only members of Podocnemididae defined from the Bartonian record of Teso de la Flecha. Jiménez Fuentes (1971) defined “*Podocnemis carbajosai*” from the anterior region of both the carapace and the plastron of a single individual (STUS 209; see figs. 1–6 in Jiménez Fuentes, 1971 and Figure 3a, b). It was diagnosed considering characters as the sinuous morphology of the anterior plastral margin, absence of contact between the gular and the pectorals, absence of overlapping between the extragulars and the entoplastron, and sinuous sutures between the epiplastra and the hyoplastra.

A few years later, also in the 1970s, de Broin (1977) performed new generic attributions for the taxa defined in Teso de la Flecha. She proposed the new combinations *N. salmanticensis* and “*Neochelys entodermica*.” Although she indicated that the generic attribution of “*Podocnemis carbajosai*” was erroneous, she proposed that this species might be more closely related to the British Eocene “*Palaeaspis*” than to *Neochelys*. Subsequently, Jiménez Fuentes et al. (1987) recognized “*Podocnemis carbajosai*” as a synonym of *N. salmanticensis*. Jiménez Fuentes, Martín de Jesús, et al. (1988) refuted the validity of “*Podocnemis entodermica*,” the material previously attributed to it being reassigned to *N. salmanticensis*. A new specimen of *N. salmanticensis* was figured in that paper, corresponding to an almost complete plastron from the type locality of the species (STUS 14359; see fig. 4 in Jiménez Fuentes, Martín de Jesús, et al., 1988; and Figure 4f).

Pérez‐García and de Lapparent de Broin (2013) recognized the validity of eight species of the genus *Neochelys*, exclusive to the European Eocene record, including a new one defined in that paper. They considered *N. salmanticensis* as the only one known in the Bartonian record. Taking into account the information up to that moment available in the literature on this European genus, as well as some details based on scarce unpublished specimens from the STUS collection, it was characterized in relation to the other *Neochelys* species by: elliptical and longer than wide carapace; peripherals 1 at least as long as wide; nuchal at least as wide as long; nuchal maximal width at least twice that of the anterior margin; seven neurals; marginals 1 less than two times wider than long; marginals 1 overlapping more than one‐third of the latero‐anterior nuchal margin; marginals 1 overlapping more than the 30% of the peripherals 1 width; straight vertebral 1 lateral margins, anteriorly diverging; vertebral 5 not being the widest of the vertebral series; subrounded anterior plastral lobe; entoplastral length less than two times its distance to the pectoral–abdominal sulcus; rounded posterior plastral lobe lateral margins; gular–extragulars complex showing the following exclusive configuration: gular wider than each extragular, extragulars not contacting or in contact with the entoplastron, relatively narrow gular–pectorals contact or absence of contact due to the presence of a short medial contact of the humerals; carapace length greater than 35 cm.

Although remains of podocnemidid turtles from the Duero Basin found in fossil sites outside the *N. salmanticensis* type horizon was cited as attributable to that species (i.e., material from Lutetian sites of the Zamora Province, see Jiménez Fuentes, 1968, 1977), subsequent works indicated that the specific attribution was doubtful (see Jiménez Fuentes, 2003, 2007). Pérez‐García et al. (2013) supported this last proposal, pointing out that the information available on the intraspecific variability of *N. salmanticensis* is very limited, and that a detailed study of this species is necessary to analyze the diversity of Podocnemididae recorded in that Basin. Considering the relatively limited material from the type locality and horizon of *N. salmanticensis* so far published, and also the information available on the representatives of the genus, Pérez‐García et al. (2017) supported that both “*Podocnemis carbajosai*” and “*Podocnemis entodermica*” correspond to synonyms of *N. salmanticensis*. The study carried out here confirms this synonymy.

## 3 MATERIALS AND METHODS

As indicated above (see Section 1), the first‐hand study of the abundant collection of shells elements of *N. salmanticensis*, from the type locality and horizon of the species (i.e., the Bartonian outcrops of the adjacent municipal terms of Cabrerizos and Aldealengua, in the Spanish Salamanca Province), deposited in the Collection of Fossil Vertebrates of the Duero Basin (Sala de las Tortugas) of the Universidad de Salamanca, is performed here. All previously published remains and a selection of numerous unpublished specimens is considered (about 150 specimens, see Figures 2, 3, 4, 5, 6, 7, 8), which allowed us to characterize in detail the shell of this taxon, as well as to evaluate character states that could be related to individual, ontogenetic, or sexual variability.

Comparisons with the shells of all other species currently recognized for the genus *Neochelys* are performed, based primarily on the first‐hand study of almost all hitherto documented individuals of most of those taxa, as well as by the consideration of all shell remains of any representative of this genus so far documented in the literature (see Cadena, 2015; Pérez‐García & de Lapparent de Broin, 2013, 2015, and references therein). Thus, a detailed and updated diagnosis for *N. salmanticensis* is proposed.

**FIGURE 10:**
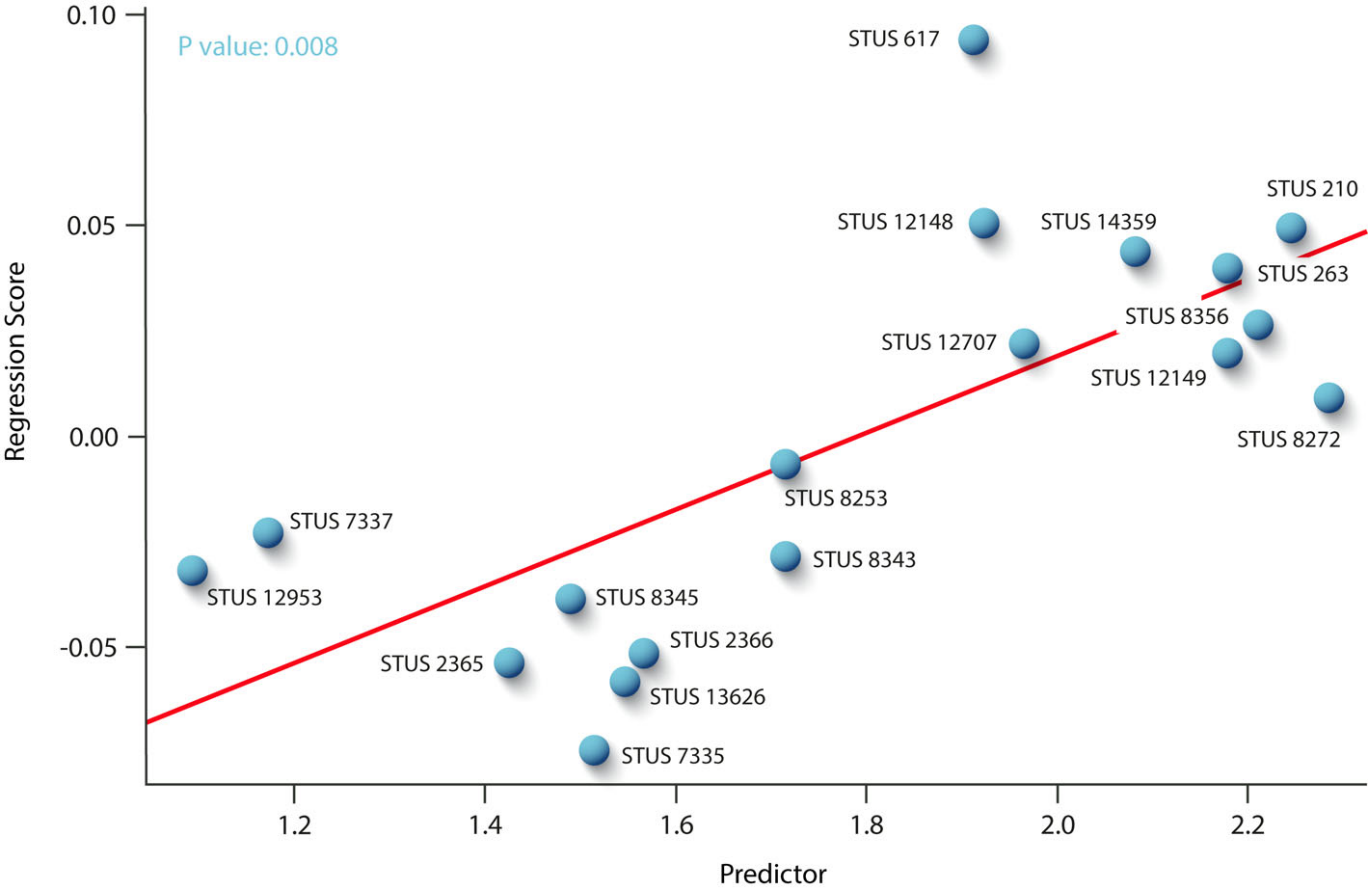
Linear regression between Procrustes coordinates (dependent variable) and logged‐centroid size values (independent variable) of the xiphiplastron of the Spanish podocnemidid turtle *Neochelys salmanticensis* from the Bartonian (middle Eocene) of Cabrerizos (Salamanca Province)

A quantitative analysis through the two‐dimensional geometric morphometric method (GMM) was performed to analyze the morphological variation present in the posterior plastral lobe of *N. salmanticensis* (Figures 9 and 10). Specifically, it has been evaluated considering the region of the xiphiplastron that involves the anal notch and the xiphiplastral processes, since they are the areas identified as sexually dimorphic in extant podocnemidid taxa (Páez et al., 2012, 2013; Sepúlveda‐Seguro et al., 2020). The two‐dimensional GMM has been followed according to the protocol proposed by Zelditch et al. (2012). The selection of landmarks was based on their ability to represent the geometric forms (Larson, 2002; Murta‐Fonseca & Fernandes, 2016). In addition, semilandmarks were added to capture the shape along their curves (Gunz & Mitteroecker, 2013). In this context, two landmarks (one on the posterior‐most point of the left xiphiplastral process and the other on the posterior‐most point of the right xiphiplastral process) and eight semilandmarks (located on the plate outline between both landmarks) were employed for the anal notch region of *N. salmanticensis*. Scale bars were used to scale each digitized specimen. All of them were digitized using the tpsDig v.2.16 software (Rohlf, 2010, 2015), and all the steps of the data collection were performed by the same operator to avoid interobserver variation in the data (Fruciano, 2016). All sets of landmarks were scaled, translated, and rotated, using Generalized Procrustes Analysis (GPA; Rohlf & Slice, 1990; Zelditch et al., 2012). This procedure was performed in R v. 4.0.1 (R Core Team, 2020) through the function *gpagen* in *geomorph* (Adams et al., 2021). The semilandmarks were slid using bending energy (Gunz & Mitteroecker, 2013), since it is the method that best fits with the nature of the study sample. For the study of the shape variation, the main maximal axes were described using the Principal Component Analysis (PCA), obtained both from the Procrustes coordinates and from the shape data corrected for allometry. However, in this case, only the former (i.e., the PCA of the Procrustes coordinates) has been included (Figure 9), since the allometric factor does not influence significatively in the shape variation of the study sample (see Section 5.1 of the Discussion for more details). The number of principal components (PCs) to evaluate in each PCA was selected through the broken‐stick method (Jackson, 1993). The PCA was computed using the function *gm.prcomp* of the geomorph package. The allometry was statistically tested through the logarithm to base 10 of centroid size (log [CS]) values (independent variable) of the specimens of each dataset against the Procrustes shape coordinates (dependent variable). The allometric regression was performed by the function *procD.lm* of the *geomorph* R package.

## 4 SYSTEMATIC PALEONTOLOGY

Pleurodira Cope, 1864.

Pelomedusoides Cope, 1868.

Podocnemidoidea Cope, 1868.

Podocnemididae Cope, 1868.

Erymnochelyinae de Broin, 1988.

*Neochelys* Bergounioux, 1954.

*Neochelys salmanticensis* (Jiménez Fuentes, 1968).

(Figs. 2, 3, 4, 5, 6, 7, 8).

### 4.1 Synonymy

*Stereogenys salmanticensis* Jiménez Fuentes (1968, 1970a, 1970b, 1970c).

*Podocnemis entodermica* Jiménez Fuentes (1970c).

*Podocnemis carbajosai* Jiménez Fuentes (1971).

“*Podocnemis*” *carbajosai* de Broin (1977), Jiménez Fuentes (1986).

*Neochelys entodermica* de Broin (1977).

*Neochelys salmanticensis* de Broin (1977), Jiménez Fuentes (1988), Jiménez Fuentes, Mulas Alonso, et al. (1988), Pérez‐García and de Lapparent de Broin (2013), Cadena (2015), Pérez‐García and de Lapparent de Broin (2015), Pérez‐García et al. (2017), Ortega et al. (2022).

“*Stereogenys*” *salmanticensis* Jiménez Fuentes (1982).

“*Neochelys*” *salmanticensis* Jiménez Fuentes (1985, 1986).

### 4.2 Type material

The holotype, STUS 180, corresponding to the anterior region of a plastron, is the only valid type material of the species (Figure 4a). A second specimen was attributed to this species in the paper where it was defined: the isolated entoplastron STUS 124 (see Jiménez, 1968). However, it did not come from the Bartonian but from the Lutetian, and not from the type area but from Corrales (in the Spanish Zamora Province instead of in the Salamanca Province), where this species is not currently identified (see Ortega et al., 2022, and references therein). Therefore, although Jiménez Fuentes (1970a, 1970b, 1970c) subsequently alluded to that second specimen as a paratype of the species, that is not correct. Jiménez Fuentes (1970a, 1970b, 1970c) attributed new specimens from the locality and type area of *N. salmanticensis* to the species, considering them as new paratypes. However, and following the International Code of Zoological Nomenclature, they cannot be recognized as type specimens since they were not cited in the original publication (in fact, they were subsequently found).

### 4.3 Other specimens attributed to the species

About 1,200 specimens inventoried, from the type area and horizon, deposited in the Collection of Fossil Vertebrates of the Duero Basin (Sala de las Tortugas) of the Universidad de Salamanca (Salamanca, Spain). A selection of the most relevant remains for the detailed characterization of the shell of this species, including the scarce previously documented specimens (9 specimens; see Section 2) and numerous previously unpublished fossils (137 specimens), from the Bartonian of Teso de la Flecha and Caenes (Cabrerizos), are presented here (see Figures 2, 3, 4, 5, 6, 7, 8).

### 4.4 Type locality and horizon

Bartonian (middle Eocene) outcrops belonging to the adjacent municipal terms of Cabrerizos and Aldealengua (Salamanca Province, Duero Basin, Castile and Leon, central Spain), the holotype having been found in an area called Teso de la Flecha (in Cabrerizos; Figure 1; for a detailed and updated geological context see Ortega et al., 2022, and references therein).

### 4.5 Emended diagnosis

*Neochelys salmanticensis* is the only representative of the genus *Neochelys* that reaches a carapace length of 50 cm. It can be defined by the following character combination: elliptical carapace; notched anterior carapace margin; sub‐equal in length and width, slightly wider than long, or slightly longer than wide nuchal; seven neurals; as wide as long or slightly wider than long second and third neurals; slightly wider than long suprapygal; slightly longer than wide pygal; slightly longer than wide first peripheral; lyre‐shaped first vertebral; substraight first vertebral posterior margin; subequal width of the second and third vertebrals, both being the widest of the vertebral series; as wide as long or slightly wider than long first marginal; anterior plastral lobe wider than the posterior; subrounded anterior plastral lobe; subrounded lateral margins of the posterior plastral lobe; anal notch width two times less than the maximum xiphiplastral width; anal notch 4–6 times wider than long; relatively long overlap of the gular on the entoplastron, reaching at least a third of the length of this plate; presence or absence of gular‐pectorals contact.

## 5 DISCUSSION

### 5.1 Intraspecific variation

#### 5.1.1 Individual variation

A relatively high morphological variability has been recognized for the shell of all *Neochelys* species represented by several individuals (de Broin, 1977; Cadena, 2015; Pérez‐García, 2015; Pérez‐García & de Lapparent de Broin, 2013, 2015), interpreted as intraspecific variation (i.e., individual variability, ontogeny, and sexual dimorphism). In this sense, the availability of numerous articulated and disarticulated elements of the shell of *N. salmanticensis* allows us to analyze this in detail for the adult stage (much more precisely than has been done for any other *Neochelys* species, considering the much larger number of remains for the taxon analyzed here). Thus, new information regarding the variability or stability of morphological characters of both the carapace and plastron of this so far poorly known representative is provided.

The variation in the relative dimensions of the nuchal plate of *N. salmanticensis* is quite subtle, this plate being about as wide as long (Figure 2e), slightly wider than long (Figure 5f), or slightly longer than wide (Figure 3f). A remarkable variation is observed in the nuchal maximal width relative to its anterior margin, the former ranging from one and a half (Figure 5f) to more than four times (Figure 3f) greater. Likewise, the nuchal length can be about two and a half times (Figure 2e) or three times (Figure 5f) the medial margin of the marginals. The most obvious variation of the nuchal plate is that related to the degree of development of the notch of its anterior margin, which can be very subtle (Figure 5g) or clearly marked (Figure 5o). The latero‐posterior margins also varies in some specimens, being substraight (Figure 5f) or curved (Figure 2i).

The neural series of most specimens is composed of seven plates (Figure 2i); however, occasionally, the sixth and seventh neurals are fused in some individuals (i.e., Figures 2g and 5an). Therefore, the presence of six elements is recognized as an anomaly. The first neural presents the greatest variation in the neural series among the *N. salmanticensis* individuals (Figures 2, 3, and 5). This plate is always characterized as longer than wide; however, the ratio between the length and the width varies among the specimens, ranging from about one and a half times longer than wide to almost two times (Figure 5ae,ac, respectively). The maximum width of the first neural can be located either in the middle region (Figure 5ac) or in the posterior half of the plate (Figure 5ag). The anterior margin of the first neural can be substraight (Figure 5ae) or anteriorly curved (Figure 5ag). A similar condition also occurs with the lateral margins (i.e., the specimens can present substraight, Figure 5aa; or curved lateral margins, Figure 5af). The morphology of the last neural remains stable in most specimens (i.e., being pentagonal and about one and a half times wider than long, Figure 5ag). However, it is heptagonal and almost as wide as long in the specimens with the fused last two neurals (i.e., Figures 2g and 5an).

Both the relative dimensions and the morphology of the suprapygal are quite stable among the *N. salmanticensis* specimens (Figure 6a–n). This plate is characterized as slightly wider than long, with a subpentagonal morphology. In this sense, the most evident variation of the suprapygal involves its posterior margins, which can be substraight (Figure 6h) or slightly curved (Figure 6n).

A notable morphological variation is identified in the pygal plate of *N. salmanticensis*, which can display substraight anterior and lateral margins (Figure 6w), or curved margins (Figure 6z). Likewise, the pygal is longer than wide, ranging from slightly longer than wide (Figure 6s) to more than one and a half times longer than wide (Figure 6x).

Relative to the carapacial scutes, morphological variation is identified in the first pair of marginals. These scutes vary in the length of their medial margin, being equivalent to the width of the complete anterior nuchal margin in some specimens (Figure 5n), but ranging to be more than three times less than it in others (Figure 5g). The first marginal can be as wide as long (Figure 2g) or slightly wider than long (Figure 2e). The first vertebral scute presents a slight variation among the specimens of the adult stage of *N. salmanticensis*. In this sense, the anterior margin of this scute can be substraight (Figure 2e) or medially concave and laterally convex (Figure 3a).

The more significant variation observed on the plastral plates of *N. salmanticensis* is located at the posterior margin of the entoplastron, which can be substraight (Figure 7ag), or slightly convex (Figure 7af).

The configuration of the gular–extragular complex is, by far, the most variable element of the plastron. The relative dimensions of the gular range from slightly longer than wide (Figure 2f) to almost two times longer (Figure 4f). The maximum width of this scute can be located in its anterior (Figure 3b) or middle region (Figure 4a), being as wide (Figure 2f) or one and a half times wider than the extragulars (Figure 4a). Likewise, the gular–extragular sulci, as well as the gular–humeral ones, can be substraight (Figure 4c) or sinuous (Figure 4a). The overlapping of the extragulars on the entoplastron has also been identified as variable in the *N. salmanticensis* specimens, with one (Figure 7ai), both (Figure 7y), or neither (Figure 7ah) of these scutes overlapping this plate. Likewise, the entoplastron can be slightly more than two times (Figure 4f) longer than the overlapping of the gular in this plate and about three times (Figure 2f). The humeral scutes of some specimens display a medial contact (Figure 2f), whereas both are separated by the gular scutes in others (Figure 4f). The length of the medial contact between the humerals is from almost the length of the gular on the entoplastron (Figure 2f) to less than half that length (Figure 2h). The humeral–pectoral sulcus can be substraight as in STUS 263 (Figure 2j) or anteriorly curved, as in STUS 180 (Figure 4a). Likewise, the most lateral point of the humeral–pectoral sulcus can be located on the postero‐lateral vertex of the epiplastron (Figure 7l), on its lateral margins (Figure 7v), or reaching the hyoplastron (Figure 4b). The pectoral scutes can overlap the anterior area of the mesoplastron (Figure 4c), being in contact with its anterior margin (Figure 2j), or not reaching this plate (Figure 4d). In most specimens, the fifth marginal scutes do not overlap the mesoplastron (Figure 4f); however, they reach this plate in some individuals (Figure 2h). Likewise, the sixth marginals overlap the lateral region of the mesoplastra, ranging from about three times less than the maximum width of this pair of plates (Figure 7ak), to more than six times less (Figure 7aj).

#### 5.1.2 Ontogeny

The availability of several specimens of different sizes (Figures 2, 3, 4, 5, 6, 7, 8) has allowed us to evaluate some morphological variations present in *N. salmanticensis* through its ontogenetic development. In addition, we provide new data regarding the ontogenetic development within the *Neochelys* genera, which is quite unknown to date, *Neochelys franzeni* being the only species in which the ontogeny has been analyzed, considering scarce characters (Cadena, 2015; Pérez‐García, 2015).

The ontogenetic stages are differentiated here based on the specimen size since most plates and scutes are invariable in morphology throughout the ontogeny of *N. salmanticensis*. Thus, two principal ontogenetic stages are identified: juveniles (Figure 2a–d) and adults (Figure 2e–j). However, due to the great size variation observed both in articulated and disarticulated remains, several ontogenetic stages (e.g., subadults) are probably represented. Nevertheless, it is complex to delimit intermediate ontogenetic stages for *N. salmanticensis* since some of the size differences can also be related to the sexual size dimorphism, as described in extant podocnemidids (e.g., Páez et al., 2013).

In this context, the vertebral scutes are one of the carapace elements that present variation among the different sizes of the *N. salmanticensis* specimens. Specifically, the length/width ratio of the second and third vertebral scutes decreases as the individuals grow. Thus, the width of the second and third vertebrals is about one and a half times their length in the juvenile stage (Figure 2a), whereas both scutes are slightly wider than long in the adults (Figure 2e). The variation of this character has been identified as clearly dependent of the ontogeny in some extant podocnemidids, as in *Podocnemis lewyana* (e.g., ZA AC 1944‐282 and MNHN.RA T0607‐1). This is also probably the case for the taxon studied herein; however, a larger sample would be necessary to confirm it with confidence. In the case of *N. franzeni*, a variation in the vertebrals throughout the ontogeny was also reported in the study of Pérez‐García (2015). There is a subtle variation for these elements among individuals of different sizes of *N. franzeni*, the second and third vertebrals being one and a half times wider than long in the largest specimens and two times wider than long in the smallest. However, as in *N. salmanticensis*, a larger sample would be necessary to confirm this character as dependent on the ontogeny.

A median ridge (i.e., a medial keel) on the neural series of the immature stage was observed both in *N. franzeni* (Cadena, 2015; Pérez‐García, 2015) and in some extant podocnemidids (e.g., *P. lewyana*, Gallego‐García & Forero‐Medina, 2014). However, this character is not present in the smaller specimens of *N. salmanticensis* (Figure 2a) since the surface of their neural series is smooth, without any type of elevation.

#### 5.1.3 Sexual dimorphism

Extant podocnemidids, as in other turtle lineages, present a wide assemblage of sexually dimorphic characters that can manifest through sexual shape dimorphism (e.g., Sepúlveda‐Seguro et al., 2020), or sexual size dimorphism (e.g., González‐Zárate et al., 2014). These variations are subtle in some species (e.g., Kuchling, 1988) or significantly marked in others (e.g., Gallego‐García & Forero‐Medina, 2014).

The characters commonly associated with the sexual shape dimorphism in extant podocnemidids involve the xiphiplastral area (e.g., Páez et al., 2012) and the presence of a concavity on the posterior region of the plastron (e.g., Gallego‐García & Forero‐Medina, 2014). In this context, in the case of *N. salmanticensis*, variations have only been observed in its xiphiplastral area, specifically, in the morphology of the xiphiplastral processes and anal notch (Figure 8). This anal notch ranges from four (Figure 4e) to six times wider than long (Figure 4f). Furthermore, this region can display a relatively pointed xiphiplastral process and a V‐shaped anal notch (Figure 8w,x) or, by contrast, a subrounded xiphiplastral process and a U‐shaped anal notch (Figure 8aw–ax). However, despite these differences, there is a wide spectrum of intermediate forms between these two morphologies, which makes it difficult to identify of two morphotypes, both through a qualitative and a quantitative framework. In this sense, the quantitative analysis shows that the first three PCs of the PCA of Procrustes coordinates collectively accounts for 78% of the total shape variance. The PC1 is principally associated with the width of the xiphiplastral process; therefore, with more positive values, the xiphiplastral process is narrower and more pointed and vice versa (Figure 9a). The shape variance kept by the PC2 of this plate concerns the degree of depth of the anal notch. In this context, a xiphiplastron with a deep anal notch is observed for the negative values and vice versa (Figure 9a). The PC3 is mainly associated with the degree of opening of the lateral margins of the xiphiplastron, being more open as positive values increase (Figure 9b). In this case, none of the axes of the PCA separately or through the combination between them differentiate two clearly morphotypes of xiphiplastra; therefore, sexual dimorphism in this area does not present significant differences. Although this region can be sexually dimorphic, the probable interaction with other types of intraspecific variation (e.g., individual or ontogenetic variation) produces a wide spectrum of morphologies, which makes it difficult to characterize of two sexual morphotypes.

Sexual size dimorphism is usually present, to a greater or lesser degree, in extant podocnemidids. Thus, this can appear as a small variation (e.g., in *Erymnochelys madagascariensis*; García, 2005; Grandidier, 1867; Kuchling, 1988) or as a considerable difference among mature specimens, one sex being much larger than the other (e.g., in *Podocnemis unifilis*, Páez et al., 2012; Troschel, 1848). In *N. salmanticensis*, both sexes appear to attain approximately the same maximum size. However, it must be considered that turtles have an indeterminate growth, which is problematic when studying extinct species due to possible confusing interactions between age and size distributions (Berry & Shine, 1980; Stamps & Andrews, 1992). Furthermore, it is common in turtles for one sex to become sexually mature at a younger age (Gibbons & Lovich, 1990; Halámková et al., 2013). Consequently, it is complex to ensure that young individuals of the larger sex of *N. salmanticensis* are not confused with older individuals of the smaller sex. In this case, due to the wide spectrum of specimen sizes, it is not possible to establish any tendency related to sexual size dimorphism. This is also evidenced by the regression, which demonstrates that the shape of the xiphiplastron of *N. salmanticensis* is not significantly influenced by the size factor (*p* > .05) (for more details see Figure 10). Therefore, the shape variation cannot be explained by differences in size among specimens.

### 5.2 General comparisons of *N. salmanticensis* with the other representatives of the genus

#### 5.2.1 Generalities of the shell

The most complete carapaces of *N. salmanticensis* allowed us to define its morphology as elliptical, as previously reported by Pérez‐García and de Lapparent de Broin (2013). This character is shared with almost all *Neochelys* species, except for *Neochelys liriae*, which has a subpentagonal carapace. In addition, contrary to all other species, the carapace of the latter is wider than long (for the comparison of this and most of the characters discussed here between the different representatives of the genus *Neochelys* see table 2 in Pérez‐García & de Lapparent de Broin, 2013). In *N. salmanticensis*, the maximum shell length is about 50 cm, whereas the maximum width is about 40 cm (Figure 2g). This also coincides with the analysis performed by Pérez‐García and de Lapparent de Broin (2013), which described the carapace length of *N. salmanticensis* as greater than 35 cm, like those of *Neochelys capellinii*, *Neochelys eocaenica*, and *Neochelys laurenti*. However, the maximum length of none of them reaches that recognized for *N. salmanticensis*, being less than 45 cm. Likewise, the length of the carapace was reported to be around 25 cm in other *Neochelys* representatives (i.e., *Neochelys arenarum*, *N. franzeni*, *N. liriae*, and *Neochelys zamorensis*).

In accordance with Pérez‐García and de Lapparent de Broin (2015), the anterior plastral lobe of *N. salmanticensis* is wider than the posterior one, as in *N. arenarum*, *N. franzeni*, and *N. liriae*. By contrast, the anterior plastral lobe is slightly narrower than the posterior in *N. capellinii*, *N. laurenti*, and *N. zamorensis*. As previously reported by Pérez‐García and de Lapparent de Broin (2013), *N. salmanticensis* shares a subrounded anterior plastral lobe with all *Neochelys* species, except *N. arenarum* and some specimens of *N. zamorensis*, which have a trapezoidal one. The lateral margins of the posterior plastral lobe in *N. salmanticensis* are rounded, as in all other *Neochelys* species, except *N. zamorensis*, which has straight margins.

#### 5.2.2 Carapace plates

The nuchal plate of most *N. salmanticensis* specimens is roughly as wide as long (Figure 5f), although it has also been found that can be slightly longer than wide (Figure 3f), or slightly wider than long in some specimens (Figure 5f). Nuchals slightly wider than long and others as wide as long are known for almost all other species of *Neochelys* (i.e., *N. arenarum*, *N. capellinii*, *N. eocaenica*, *N. franzeni*, *N. laurenti*, and *N. zamorensis*). *Neochelys liriae* is the species with a greater width/length ratio of the nuchal, being one and a half times wider than long. As in *N. salmanticensis*, variation in the nuchal maximal width in relation to that of its anterior margin can be identified in the other *Neochelys* species represented by several individuals. However, that variation is recognized as smaller than that observed in the species studied herein. Specifically, the maximal width of the nuchal ranges from two to two‐and‐a‐half times greater than the anterior margin width in *N. arenarum*, *N. eocaenica*, *N. franzeni*, *N. laurenti*, and some *N. capellinii* specimens. However, the maximum nuchal width is about one and a half times greater than the anterior margin of the plate in *N. zamorensis* and some specimens of *Neochelys capellini*. The anterior margin of the nuchal does not present significant variation in its curvature for most *Neochelys* species, being substraight in *N. arenarum*, *Neochelys fanzeni*, *N. zamorensis*, and some specimens of *N. capellinii*, *N. eocaenica*, and *N. laurenti*. It is slightly concave in some *N. capellinii, N. eocaenica*, and *N. laurenti* individuals, but without presenting a notch as well‐developed as in some specimens of *N. salmanticensis* (Figure 5n). As in *N. salmanticensis*, *N. eocaenica* also presents variation in the number of neural plates. However, the number of neurals of the latter shows a greater range of variation than that identified for *N. salmanticensis*, varying between six and eight (see fig. 20 in de Broin, 1977) instead of six and seven. In the specimens of *N. eocaenica* with eight neurals, the last of these elements is in contact with the suprapygal, whereas in the rest of the *Neochelys* taxa, the last neural does not contact with the suprapygal. Except *N. arenarum*, which has six neurals, the other *Neochelys* species (i.e., *N. capellinii*, *N. laurenti*, *N. liriae*, and *N. franzeni*) present seven neurals, as most *N. salmanticensis* specimens. The morphology of the neural series is similar in all *Neochelys* species (i.e., the first neural being rectangular; the second to penultimate, hexagonal; and the last pentagonal), except in cases where fusion of the last two neurals is present, resulting in an anomalous element. A slight variation for the relative dimensions of the neurals can be observed among the *Neochelys* representatives, especially considering the second and third ones. Thus, the second and third neurals of *N. salmanticensis* (Figure 2e), *N. arenarum*, and *N. franzeni* are as wide as long, or slightly wider than long. They are noticeably wider than long in *N. laurenti* and *N. liriae*. By contrast, the second and third neurals of *N. capellinii* are slightly longer than wide, and the second to fifth neurals of *N. zamorensis* are noticeably longer than wide. Variation in the relative dimensions of the suprapygal plate can be observed when the *Neochelys* species are compared. Thus, this element is lightly wider than long in some of them (i.e., *N. capellinii*, *N. eocaenica*, *N. laurenti*, *N. liriae*, and *N. salmanticensis*; Figure 6n), but about two times wider than long in others (i.e., *N. franzeni* and *N. zamorensis*). *Neochelys salmanticensis* coincides with some *N. franzeni* specimens in that the pygal plate is longer than wide (Figure 6z). By contrast, this plate is as long as wide in *N. liriae*, and slightly wider than long in the other representatives (i.e., *N. capellinii*, *N. laurenti*, *N. zamorensis*, and some specimens of *N. franzeni*). The relative dimensions of the first peripheral plate of *N. salmanticensis* coincide with those reported by Pérez‐García and de Lapparent de Broin (2013), which were described as slightly longer than wide (Figure 2g). This character coincides in almost all *Neochelys* species, except in *N. liriae*, which presents a wider than long first peripheral.

#### 5.2.3 Caparace scutes

As indicated above, the vertebral scutes of *N. salmanticensis* present a relatively high intraspecific variation. In this sense, the anterior margin of the first vertebral of this species can be substraight (Figure 2e), as in *N. arenarum*, or medially concave and laterally convex (Figure 3a), as in *N. liriae*, *N. franzeni*, and some *N. capellinii*, *N. eocaenica*, and *N. laurenti* specimens. In the case of *N. zamorensis* and some specimens of *N. capellinii*, *N. eocaenica*, and *N. laurenti*, the anterior region of this scute is almost straight, with anteriorly diverging margins. Likewise, as reported by Pérez‐García and de Lapparent de Broin (2013), the lateral margins of the first vertebral of the representatives of this genus can be substraight (i.e., *N. capellinii*, some specimens of *N. franzeni*) or lyre‐shaped sensu Pérez‐García & de Lapparent de Broin, 2013 (i.e., with a slight curvature; i.e., *N. arenarum*, *N. eocaenica*, *N. laurenti*, *N. liriae*, *N. salmanticensis*, *N. zamorensis*, and some *N. franzeni* specimens). The posterior margin of that vertebral can be subtraight (i.e., *N. salmanticensis*, *N. capellinii*, *N. eocaenica*, *N. liriae*, and some specimens of *N. laurenti*), concave (i.e., *N. franzeni* and some specimens of *N. laurenti*), or medially convex and laterally concave (i.e., *N. zamorensis* and some specimens of *N. franzeni*). Concerning the relative dimensions of the first vertebral, this scute is slightly wider than long in most *Neochelys* species (i.e., *N. salmanticensis*, *N. arenarum*, *N. eocaenica*, *N. franzeni*, *N. laurenti*, *N. liriae*, and *N. zamorensis*), the only exception being *N. capellinii*, where it can be slightly longer than wide, as wide as long, and slightly wider than long. As reported by Pérez‐García and de Lapparent de Broin (2013), the last vertebral is wider than the other vertebrals in *N. liriae* and *N. eocaenica*, whereas the second and third vertebrals are the widest in *N. salmanticensis* (Figure 2i), *N. capellinii*, *N. franzeni*, *N. laurenti*, and *N. zamorensis*. Variation is observed in the relative dimensions of the first marginal scute when the *Neochelys* species are compared. In this sense, this scute can be as wide as long (i.e., some specimens of *N. salmanticensis*, Figure 2g), slightly wider than long (i.e., *N. franzeni* and some specimens of *N. salmanticensis*, Figure 2e), about one‐and‐a‐half times wider than long (i.e., *N. arenarum* and some *N. laurenti* specimens), two times wider than long (i.e., *N. capellinii*, *N. franzeni*, and some *N. laurenti* specimens), two‐and‐a‐half times wider than long (i.e., *N. eocaenica*), and three times wider than long (i.e., *N. liriae*). Likewise, the medial margin of that scute is longer than the lateral one in *N. capellinii*, *N. eocaenica*, *N. franzeni*, *N. liriae*, *N. zamorensis*, and some *N. laurenti* specimens. By contrast, the medial and lateral margins are subequal in *N. arenarum*, *N. liriae*, *N. salmanticensis* (Figure 2e), and some specimens of *N. laurenti*. The length of the overlapping of the first pair of marginals on the nuchal plate varies among the different *Neochelys* species. Thus, the nuchal length can be about two‐and‐a‐half times the medial margin of the marginals (i.e., some *N. salmanticensis* specimens; Figure 2e), about three times (i.e., *N. arenarum*, *N. liriae*, and some *N. laurenti* and *N. salmanticensis* specimens; Figure 5f), about five times (*N. capellinii*, *N. eocaenica*, *N. franzeni*, and *N. zamorensis*), and six times (i.e., some *N. laurenti* specimens).

#### 5.2.4 Plastral plates

As previously reported by Jiménez (1968), *N. salmanticensis* share with other species a rhomboidal, slightly wider than long entoplastron (Figure 7x–ai). The more significant variation observed in this plate among the other *Neochelys* species concerns the posterior margins, which can be substraight (i.e., *N. arenarum*, *N. eocaenica*, *N. franzeni*, *N. liriae*, *N. zamorensis*, and some specimens of *N. laurenti* and *N. salmanticensis*, Figure 7ag), or slightly convex (i.e., *N. capellinii* and some *N. laurenti* and *N. salmanticensis* specimens, Figure 7af). As reported by Pérez‐García and de Lapparent de Broin (2013), the entoplastral length can be two times or a slightly more than two times its distance to the pectoral–abdominal sulcus (i.e., *N. arenarum*, *N. liriae*, and some specimens of *N. capellinii*, *N. laurenti*), less than two times that distance (i.e., *N. eocaenica*, *N. franzeni*, and *N. salmanticensis*), or about the same distance (i.e., *N. zamorensis*). The width of the anal notch of *N. salmanticensis* is about two times less than the maximum width of the xiphiplastra, this condition being shared with *N. franzeni*, *N. laurenti*, *N. liriae*, and *N. zamorensis*. By contrast, the width of the anal notch of *N. arenarum*, *N. capellinii*, and *N. eocaenica* is two and a half times less than the maximum width of the xiphiplastra. Likewise, the anal notch of *N. salmanticensis* ranges from four to six times wider than long (Figure 4e,f, respectively), contrasting with the condition in most *Neochelys* species, where it is between two and three times wider than long.

#### 5.2.5 Plastral scutes

The configuration of the gular and extragular complex has been described and compared among the different *Neochelys* species by Pérez‐García and de Lapparent de Broin (2013) as a combination of characters (i.e., dimensions of the extragulars in relation to the gular, degree of overlap of the gular on the entoplastron, presence and development of contact between extragulars and pectorals, and presence and development of medial contact between both humerals). However, in the present contribution, each of these characters has been compared separately, since they present a greater variation than that reported by Pérez‐García and de Lapparent de Broin (2013). In this context, the gular and extragular scutes are not only variable among the *N. salmanticensis* specimens, but also in other *Neochelys* representatives. The relative dimensions of both scutes show a wide range of variation, the gular scute being slightly wider than each extragular (i.e., *N. capellinii*, *N. franzeni*, *N. liriae*, and some *N. eocaenica* and *N. salmanticensis* specimens, Figure 4a); the width of these scutes can be subequal (i.e., some specimens of *N. eocaenica* and *N. salmanticensis*); but the extragulars can also be the widest, being slightly wider than the gular (*N. arenarum* and *N. laurenti*), or about two times wider (*N. laurenti* and *N. zamorensis*). Likewise, the entoplastron can be about two times longer than the overlapping of the gular in this plate (i.e., *N. arenarum*, *N. eocaenica*, *N. franzeni*, and some *N. arenarum* and some *N. salmanticensis* specimens), two‐and‐a‐half times (i.e., *N. franzeni*), three times (i.e., *N. capellinii*, *N. liriae*, and some *N. arenarum* and other *N. salmanticensis* specimens), four times (i.e., *N. capellinii* and *N. laurenti*), and six times (i.e., *N. laurenti*). In addition, as observed in *N. salmanticensis*, the overlapping of the extragulars on the entoplastron is also variable among the *Neochelys* species, with one (i.e., some specimens of *N. arenarum*), both (i.e., some specimens of *N. arenarum*, *N. laurenti*, and *N. zamorensis*), or none (i.e., *N. capellinii*, *N. eocaenica*, *N. franzeni*, and *N. liriae*) of these scutes overlapping this plate. Some species do not present gular–pectoral scute contact due to the medial contact of the humerals (i.e., *N. capellinii*, *N. laurenti*, *N. liriae*, and some *N. arenarum* and some *N. salmanticensis* specimens), whereas a contact between both scutes is present, which can be short (i.e., *N. zamorensis* and some *N. arenarum*, *N. eocaenica*, *N. salmanticensis* specimens) or long (i.e., *N. franzeni* and some *N. eocaenica* and some *N. salmanticensis* specimens).

## 6 CONCLUSIONS

The Eocene *Neochelys* is the best represented podocnemidid in the European record, considering both its diversity and the number of remains attributed to it. Its record spans several countries of this continent, having been found in fossil sites from the lower to the upper Eocene. *Neochelys salmanticensis* is the youngest of the eight known species for this genus, being the only one defined at post‐Lutetian levels. Thus, it comes from various Bartonian (middle Eocene) outcrops in the area of the Cabrerizos and Aldealengua municipalities, in the Spanish Salamanca Province. Although the species was defined several decades ago (Jiménez, 1968), and abundant material attributable to it has been found in its area and type horizon (including more than 1,200 specimens inventoried at the Collection of Fossil Vertebrates of the Duero Basin (Sala de las Tortugas) of the Universidad de Salamanca), the information about it was so far very limited. In this sense, a valid diagnosis for it was not available.

A detailed study of the *N. salmanticensis* material deposited at the above mentioned collection has been carried out. Since all *Neochelys* species are known from shell remains, a selection of nearly 150 specimens corresponding to complete to partial shells, and also to isolated or articulated plates, is here presented and discussed, including the nine previously documented fossils, and numerous to date unpublished specimens. As a result, the detailed characterization of the shell of this species, relative to that of the other known species, has been carried out. Furthermore, given the abundance of remains, several aspects related to its intraspecific variability have been documented. Thus, characters affected by individual variation are recognized but, in addition, variation in other characters as related to both ontogeny and sexual dimorphism is evaluated. In this way, and thanks to this abundant availability of specimens, the shell of *N. salmanticensis* has been here characterized in a much more precise way than that of any other European podocnemidid turtle. Thus, future work will allow us both to describe with more precision the skeleton of this species (considering cranial and appendicular elements), and to evaluate the diversity of podocnemidids in the Spanish Eocene Duero Basin, accurately characterizing the other forms recorded there.

## AUTHOR CONTRIBUTIONS

**Adán Pérez‐García:** Conceptualization; formal analysis; investigation; methodology; project administration; validation; writing – original draft; writing – review and editing. **Andrea Guerrero:** Formal analysis; investigation; methodology; software; writing – original draft; writing – review and editing. **Santiago Martín de Jesús:** Data curation; investigation; resources; writing – review and editing. **Francisco Ortega:** Funding acquisition; validation; writing – review and editing.
